# Analysis of Bound Form Terpenes in Different Agricultural Byproducts

**DOI:** 10.3390/molecules30204077

**Published:** 2025-10-14

**Authors:** Duyen Bui, Achyut Adhikari, Witoon Prinyawiwatkul, Zhimin Xu

**Affiliations:** School of Nutrition and Food Sciences, Louisiana State University, Baton Rouge, LA 70803, USA; dbui14@lsu.edu (D.B.); acadhikari@agcenter.lsu.edu (A.A.); wprinya@lsu.edu (W.P.)

**Keywords:** terpenes, bound terpenes, peels, hydrolysis, fruits, plants

## Abstract

Traditional sample preparation for terpene analysis includes distillation, solvent extraction, and solid phase extraction and is followed by using gas chromatography with a mass spectrometer (GC-MS) to complete identification and quantification. The preparations rely on the volatility and low polarity of terpenes which exist in free form. However, terpenes in bound form are still largely retained in the extracted residues because, by binding with sugar moiety, they have high polarity and water solubility and low volatility. In this study, distributions and profiles of free and bound form terpenes in different fruit and crop byproducts were evaluated by using different extraction media followed by acid hydrolysis. The acid hydrolysis significantly broke down the binding between terpene and sugar moiety and freed the bound terpene. The concentration of bound terpenes in fruit peel or corn silk was much higher than that of originally existing free terpenes. For example, the terpene concentration in watermelon peel increased from 47.0 to 101 μg/g after hydrolysis. The profile of bound terpenes was also more diverse than that of free terpenes. Among the three extraction media, water, ethanol, and acetone, acetone was the best media to extract bound terpenes with over one and a half times higher total bound terpene extraction yield than ethanol or water extract. The findings of this study explored the bound form terpenes in agricultural products which are usually underexplored in current terpene research. It also demonstrated an effective sample preparation and approach for determining bound terpenes in plants. This study could be an initiating effort and work to assist in exploring rarely mindful bound terpenes in foods and plants. The odorless nature and high stability and water solubility of bound terpenes could provide them a great advantage over free terpenes in various applications requiring neutral scent.

## 1. Introduction

Terpenes are the largest and most diverse group of aromas and are mainly found in plant sources such as fruits, vegetables, herbs, tea leaves, etc., as plant secondary metabolites [1,2,3]. They are all structured through a basic unit, isoprene, C_5_H_8_ with a general formula (C_5_H_8_)n. Based on the number (n) of the basic unit from 1 to 8, they are classified as hemiterpene, monoterpene, sesquiterpene, diterpene, sesterpene, triterpene, tetrapene, and polyterpene, respectively [4]. Their oxygen-containing derivatives are named as terpenoids. For terpenes with a number of isoprene 3 or less, they are very volatile and offer different aromatic scents, which play a crucial ecological role in pollination, plant–plant signaling, or against pathogens and herbivores [4]. All terpenes are often extracted from plants using various methods such as steam distillation, solvent extraction, or cold press to form essential oil. Recently, green or solvent-free extraction technologies such as supercritical CO_2_, microwave-assisted, and enzyme-assisted methods have been applied for essential oil extraction to improve recovery efficiency and ensure safety for application, especially for food preservation. Food microbial spoilage is one of the main issues for food quality loss; it was reported that the synergistic effect of essential oil constituents enhanced the inhibitory capability against the spoilage and extended food shelf life [5]. Also, many essential oils have antibacterial potential against the contamination of phytopathogenic bacteria which usually damage the quality of cereals, pulses, fruits, and vegetables [6].

In recent years, the mechanisms of bioactive properties of terpenes, particularly their antimicrobial and health-promoting effects, have gained more attention. For example, α-pinene and linalool have demonstrated antimicrobial activity against *Escherichia coli* and *Staphylococcus aureus* through disruption of bacterial membranes, which is associated with the intermolecular hydrogen-bonding capacity of terpene [7,8,9,10]. Similarly, thymol and carvacrol which are found in thyme and oregano exhibit strong antibacterial, antifungal, and anti-inflammatory activities which make them useful for food preservation and therapeutic applications [11,12,13]. Normally, Gram-positive bacteria are more vulnerable to terpenes than Gram-negative bacteria because the structure of the cell wall of Gram-positive bacteria is hydrophilic, whereas the wall of Gram-negative bacteria mainly consists of lipopolysaccharides, which prevent the dispersion of terpenes [13]. In addition to antimicrobial activities, terpenes, such as limonene, have antioxidant and anti-obesity effects via modulation of lipid metabolism and oxidative stress [14,15]. These multifunctional properties emphasize the promise of terpenes as natural bioactive agents for applications in food, pharmaceuticals, and health-promoting supplement industries. These broad bioactivities also indicate their important roles as sustainable alternatives to synthetic additives, which can align with consumer demand for natural and clean-label products.

Most terpenes discussed in the literature, research, and applications are free form terpenes or free terpenes in essential oil, which are volatile, aromatic, easily noticeable, and directly contribute to plant aroma and flavor profiles. However, there is also a large group of terpenes existing in bound form. Bound terpenes are conjugated with one or more sugar moieties to form glycosidic compounds that are non-volatile or odorless and water-soluble [16]. They are usually underexplored and overlooked in most terpene studies. Only a few studies related to grape wine flavor quality have investigated bound terpenes. During winemaking, the fermentation process helps to increase acidity which can hydrolyze the glycosidic bonds and release free form terpenes that contribute to the wine aroma [17,18]. Therefore, bound form terpenes are considered as wine flavor reserve and significantly impact the sensory property of the final product. Bound form terpenes are typically found in plant tissues, where they act as precursors that can also be enzymatically released under certain conditions [19]. As the presence of sugar moiety in bound terpenes confers high polarity and water solubility, it significantly reduces their volatility and renders them odorless compared to their free form terpenes. The sample preparation for free form terpenes analysis is no longer suitable to prepare bound form terpenes for analysis. Instead, hydrolysis employed to release aglycones or free form terpenes is required when GC-MS is applied to carry out identification and quantitation.

However, the exploration of bound terpenes in agricultural products and byproducts, especially byproducts, could generate a novel utilization of byproducts and increase the overall economic value of agricultural products. Therefore, the objective of this study was to determine the bound terpenes in inedible fruit peels from honeydew, watermelon, and pineapple and crop byproduct corn silk to explore bound terpenes in plants. As mentioned, the chemical property of bound terpenes is completely different from free form terpenes; in this study, highly polar media, water and ethanol, and moderately polar media, acetone, were applied and compared with their capability of extracting bound terpenes from plant tissues. After the acid hydrolysis for converting bound terpenes to free terpenes, they were determined by solid phase microextraction (SPME) coupled with gas chromatography with mass spectrometer (GC-MS). The development and optimization of extraction media and analytical protocol provided an effective approach in the determination of bound terpenes in plants. It could initiate more studies on bound terpenes which have not been given attention in present terpene research. Meanwhile, the information of profiles and concentrations of bound terpenes in agricultural byproducts holds promise for discovering novel applications and sustainable strategies for managing organic wastes. This research also highlights the importance of investigating bound terpenes because they present as reservoirs of aroma and bioactivity for plants and plant foods. They could play an important role in impacting sensory property, food preservation, and health-promoting functions as free form terpenes.

## 2. Results and Discussion

### 2.1. Extraction Yields of Studied Byproducts Using Different Extraction Media

In this study, three common fruit peels, honeydew, watermelon, and pineapple peels, and crop byproduct, corn silk, are selected as the representative agricultural byproducts for studying the distribution and profile of bound terpenes in plants. Although they are traditionally considered as inedible byproducts, recent studies found that they are rich sources of fibers, minerals, and even antioxidant phenolics. These constituents could have great potential to be used as functional food ingredients to enhance the nutritional value of food products.

For example, the liquid fraction of honeydew peel contains not only a high content of minerals but also antioxidant polyphenolics and carotenoids, such as flavones and hydroxycinnamic acids, lutein, and β-carotene [20]. Also, watermelon peel is rich in various minerals and vitamins and can be made as dried power extract for food applications to reduce environmental burden [21]. Similar to the two fruit peels, most essential nutrients, including calcium, potassium, vitamin C, carbohydrates, and dietary fiber, are present in pineapple peel. Some studies also found the pharmacological properties of pineapple peel, including anti-parasitic effects, constipation relief, etc. It could be derived from food products, such as jelly and pickles, and increase its economic value [22].

Corn silk is usually used for making corn silk tea in South Asian countries as it has a pleasant and fresh smell [23]. It is also considered an important herb used to treat many diseases, acting as a diuretic agent in hyperglycemia reduction, as an anti-depressant, and treating anti-fatigue or urinary problems [24]. Terpene and terpenoids, which contribute to the smell, have antioxidant activity, hyperglycemia alleviation, and fatigue reduction [24].

For extraction of free terpenes or essential oil in plants, solvent, solvent-based extraction, hydro-distillation, and steam distillation methods are widely applied [25]. Recently, microwave and supercritical fluid technology was used to assist the efficiency of solvent extraction [25]. However, for determination of terpenes in plants, solvent-based extraction is usually adapted to prepare samples before instrument analysis. Based on different chemical properties of terpenes, polar solvent such as ethanol was applied for extraction of polar terpenes, while non-polar solvent hexane was used for isolation of non-polar terpenes which usually consist of more than four basic units [26]. For bound terpene, its polarity is much higher than free form terpene because it consists of sugar moiety and has strong hydrophilicity. However, the part of terpene in bound terpene is hydrophobic. Therefore, the polarity of bound terpene could be between sugar and free terpene. In this study, polar media, water and ethanol, and intermediately polar solvent acetone were used to prepare the bound terpene extracts. Their efficiencies of bound terpene extraction were compared by differences in the initial level of free terpenes and the final level of terpenes after acid hydrolysis.

The total extraction yields of the three media in extracting different plant tissues are listed in Table 1. The yield of water or ethanol extraction was higher than that of acetone extraction in all the samples, especially in fruit peel samples. It may result from the high level of water-soluble materials, such as sugar and minerals, in fruit peel [20]. Some of them were reluctantly dissolved in acetone. Acetone is a common and safe solvent for food applications, such as vegetable oil extraction. Its intermediate polarity could provide more diversity in extracting different polarity compounds. It has been widely applied in extraction of natural plant materials, such as phenolics, carotenoids, etc.

### 2.2. Concentrations and Profiles of Major Terpenes in Water, Ethanol, and Acetone Extracts Before and After Hydrolysis

The acid hydrolysis significantly increased the total terpene concentration in all extracts (Table 2, Table 3 and Table 4). It confirmed the presence of glycosidically bound terpenes in these byproducts. Overall, acetone extract always had the highest concentration of terpenes, which were released and increased by bound terpenes in extract after acid hydrolysis. Among the samples, the bound terpenes in honeydew and pineapple peels were not readily extracted by either water or ethanol. This may be caused by their high soluble sugar content and firm texture. Compared with acetone, water and ethanol were less capable of penetrating the cellulose matrix and extracting bound terpenes in the matrix. However, water was better in corn silk extraction, while both water and ethanol had similar efficiency in watermelon extraction. The increase in terpene concentration in all the samples after acid hydrolysis indicated that bound terpenes could be present at a significant amount in many plant tissues and should be analyzed in the research related to plant terpenes.

There were several terpenes, such as epicubebol, calamenene, aristol-1(10)-en-9-ol, (-)-isolongifolol methyl ether, copaene, caryophyllene, γ-himachalene, α-calacorene, and geranyl acetone, which existed or increased in the extracts of honeydew peel, watermelon peel, pineapple peel, and corn silk after hydrolysis (Table 2, Table 3 and Table 4), especially in acetone extracts. Epicubeol and calamenene were commonly in honeydew, watermelon, and pineapple peel extracts, while aristol-1(10)-en-9-ol was found in honeydew, watermelon, and corn silk. Copaene or α-Copaene was present in honeydew and pineapple. In previous studies, it was reported that copaene was widely distributed in essential oils and recognized as a major constituent of the copaiba tree oleoresin. It exhibits notable antimicrobial activity, particularly against foodborne pathogens, such as *Staphylococcus aureus*, *Escherichia coli*, *Bacillus cereus*, and *Shigella bogdii*, where it can disrupt their cell membranes, increase permeability, suppress biofilm formation, and maintain good biosafety at effective concentrations [32]. Epicubebol, a sesquiterpenoid alcohol reported in *Cryptomeria japonica* and *Streptomyces griseus*, has been shown to modulate immune responses by inducing dendritic cell differentiation and promoting IL-10-producing regulatory T cells, suggesting an important role in immune tolerance and anti-inflammatory regulation [33]. In addition, epicubebol, abundant in corn silk, showed antifungal and antioxidant activity which may contribute to the health benefit of corn silk tea [29]. Calamenene has been identified in *Camellia sinensis* and *Tilia* spp., where it functions in plant wound defense and exhibits antimicrobial, anti-inflammatory, and antioxidant properties, including membrane disruption, inhibition of inflammatory mediators, and promotion of wound healing [34]. Similarly, caryophyllene found in pineapple and watermelon peels has important roles in plants, inhibiting microbial growth, being a natural enemy of herbivores, and being an indirect defense mechanism. It is well-known for antimicrobial activity against *Staphylococcus aureus* and *Escherichia coli* [27,35]. It was noticed that original and generated free form terpenes are not as stable as bound terpenes. Some of them could be oxidized or degraded during sample preparation. For example, the concentration of epicubebol in pineapple and corn silk water extracts decreased after acid hydrolysis.

**Table 3:** Retention times (RT), retention index (RI), library retention index (LRI), concentrations (μg/g of extract) and odor perception of major terpenes in watermelon peel extracts without (before) and with (after) acid hydrolysis.

			Water		Ethanol		Acetone		Odor
Compound	RI	LRI	Before	After	Before	After	Before	After	Perception
Caryophyllene	1422	1419	-	-	-	13.9 ± 0.3	-	14.1 ± 0.4	Woody ^1^
γ-Himachalene	1481	1477	-	-	-	-	-	14.7 ± 0.3	Cedar, woody ^2^
Epicubebol	1496	1493	-	-	13.7 ± 0.3 ^a^	-	14.8 ± 0.3 ^a^	37.3 ± 0.4 ^b^	Pleasant aroma, herbal ^3^
Calamenene	1518	1523	15.8 ± 0.3 ^a^	16.8 ± 0.4 ^a^	13.6 ± 0.3 ^a^	17.6 ± 0.3 ^a^	13.9 ± 0.3 ^a^	17.1 ± 0.3 ^a^	Herb spice, minty ^4^
α-Calacorene	1541	1542	-	-	-	20.6 ± 0.3 ^a^	-	17.8 ± 0.3 ^a^	Dry-woody ^5^
Aristol-1(10)-en-9-ol	1623	1642	-	-	-	-	18.3 ± 0.3	-	Woody, floral ^6^
(-)-Isolongifolol, methyl ether	1648	1645	17.9 ± 0.3 ^a^	19.3 ± 0.4 ^ab^	-	13.9 ± 0.3 ^b^	-	-	Woody ^7^
Total			33.7	36.1	27.3	66.0	47.0	101.0	

Data in the same row with different letters indicate there is a significant difference between them. ^1^: [35] Zhang et al., 2024; ^2^: [36] Uehara et al., 2017; ^3^: [28] Kirimer et al., 1996; ^4^: [29] Hutchings et al., 2025; ^5^: [37] Van et al., 2013; ^6^: [30] Jiang et al., 2024; ^7^: [31] Sant’Anna et al., 2007.

**Table 4:** Retention times (RT), retention index (RI), library retention index (LRI), concentrations (μg/g of extract) and odor perception of major terpenes in pineapple peel extracts without (before) and with (after) acid hydrolysis.

			Water		Ethanol		Acetone		Odor
Compound	RI	LRI	Before	After	Before	After	Before	After	Perception
Copaene	1375	1376	-	-	-	13.9 ± 0.3 ^a^	-	13.9 ± 0.3 ^a^	Woody, spice ^1^
Caryophyllene	1422	1419	17.2 ± 0.4 ^a^	-	-	-	14.6 ± 0.3 ^a^	16.3 ± 0.4 ^a^	Woody ^2^
Geranyl acetone	1459	1453	-	-	-	-	13.8 ± 0.3 ^a^	14.4 ± 0.4 ^a^	Violet, rose, fruity ^3^
γ-Himachalene	1481	1477	-	-	-	-	13.8 ± 0.3 ^a^	14.6 ± 0.4 ^a^	-
Epicubebol	1496	1493	23.8 ± 0.3 ^a^	15.2 ± 0.3 ^b^	-	14.1 ± 0.3 ^b^	18.1 ± 0.3 ^c^	18.1 ± 0.4 ^c^	Pleasant aroma, herbal ^4^
Calamenene	1518	1523	-	-	-	-	-	14.9 ± 0.4	Herb spice, minty ^5^
α-Calacorene	1541	1542	-	-	-	-	14.9 ± 0.3 ^a^	15.9 ± 0.3 ^a^	Dry-woody ^6^
Total			41.0	15.2	0	28.0	75.2	108.1	

Data in the same row with different letters indicate there is a significant difference between them. ^1^: [27] Hu et al., 2019; ^2^: [35] Zhang et al., 2024; ^3^: [29] Hutchings et al., 2025; ^4^: [28] Kirimer et al., 1996); ^5^: [38] Bonikowski et al., 2015; ^6^: [37] Van et al., 2013.

### 2.3. Odors of Terpenes in the Byproduct Extracts and Their Relationships with Various Functions

These sesquiterpenes, serving as defense-related metabolites in plants as discussed above, partially rely on their unique aroma. For example, γ-himachalene and α-calacorene detected in watermelon and corn silk extracts have the characteristic smell of cedarwood and vetiver oils [36,37]. They have ecological roles including antimicrobial protection and interaction with pollinators [39]. Also, α-calacorene has been associated with antimicrobial activity against Gram-negative bacteria as well as antioxidant properties, highlighting its potential utility in the development of new antibacterial and therapeutic agents [39]. However, aristol-1(10)-en-9-ol and (-)-isolongifolol methyl ether appeared primarily in honeydew and watermelon peel extract after acid hydrolysis, which suggested that they occurred in conjugated forms within the plant matrix. These compounds are usually released during tissue disruption or processing and can enhance both plant defense and sensory quality with floral and woody smells [28,30,40]. Likewise, geranyl acetone, with fruity notes observed in pineapple, has been linked to antioxidant and antimicrobial activities [29].

From a sensory perspective, the aroma profiles of the identified bound terpenes can be classified into four dominant categories that reflect their chemical diversity and odor contributions (Table 2, Table 3, Table 4 and Table 5). The woody class includes copaene, caryophyllene, γ-himachalene, α-calacorene, aristol-1(10)-en-9-ol, and (–)-isolongifolol methyl ether. They impart characteristic dry, woody, cedar-like, or resinous notes that are often associated with plant defense and ecological signaling [27,31]. The spicy–minty class, represented by calamenene and caryophyllene, contribute to warm, pungent, and slightly cooling aromas of herbal spices [29]. Epicubebol falls within the herbal category, providing a pleasant, green, and medicinal odor that complements other volatiles in the mixture [28]. On the other hand, geranyl acetone defines the fruity–floral class, imparting violet- and rose-like nuances that are frequently linked to floral and ripe fruit perceptions [29,38].

The release of these woody, spicy, herbal, and fruity–floral flavors from bound terpenes during hydrolysis could intensify the aroma complexity initially observed in original plant tissues. Acid treatment liberates glycosidically bound terpenes, thereby transforming the aroma from subtle or muted to a more pronounced and multidimensional flavor. This process highlights the importance of bound volatile reservoirs in plant tissues, which can remain inactive until enzymatic or chemical hydrolysis occurs [41]. Such transformations have been documented in winemaking and fermented juice processing, where glycosidically bound volatiles are the key contributors to aroma development during fermentation and aging. Their release enhances overall sensory quality by introducing floral, fruity, spicy, and woody notes that would otherwise remain latent in the bound form [17]. Meanwhile, the odorless nature of bound terpene offers a significant promise for the applications in food preservation, pharmacology, and biotechnology, which restrict any scent in the products.

## 3. Materials and Methods

### 3.1. Chemicals and Plant Materials

Acetone, ethanol, and hydrochloric acid were purchased from Sigma-Aldrich (St. Louis, MO, USA). Honeydew, watermelon, pineapple, and corn silk were obtained from the local markets in Baton Rouge, LA, USA.

### 3.2. Sample Preparations and Extractions

Honeydew, watermelon, pineapple, and corn silk were thoroughly cleaned and rinsed under distilled water to remove surface contamination. Peels of honeydew, watermelon, and pineapple were removed using a fruit peeler. Then, each type of sample (300 g) was ground and separated into three groups. The ground sample was mixed with distilled water, ethanol, or acetone at a ratio of 1:1 (wt.:wt.). The mixture was blended until a homogenous and lump-free slurry was obtained. The homogenous samples were sonicated for one hour in a sonication bath (Branson 1510, 40 kHz, 70 W, Ultrasonics Corp. Danbury, CT, USA) followed by centrifugation at 5000 rpm for twenty minutes. The supernatant was filtered in a funnel with filter paper. The residue on the filter paper was repeated to carry out extraction using the same media two times. The combined supernatant was evaporated at 60 °C under vacuum until the extract was completely dried. The extraction of each media was carried out in triplicates.

### 3.3. Determination of Free and Bound Terpenes by Solid Phase Microextraction (SPME) Coupled with GC-MS

For determination of free terpenes existing in extract, 50 mg of extract was placed in a SPME vial and was re-dissolved in 2 mL of distilled water. The mixture was vortexed uniformly before SPME extraction. For determination of bound terpenes in extract, after 50 mg of extract was placed in s SPME vial, 20 μL of HCl (10 M) was added and vortexed to carry out acid hydrolysis and followed by SPME extraction.

The fiber for SPME extraction was carboxen/PDMS (polydimethylsiloxane) fiber with a 75 μm-thickness coating (Sigma-Aldrich, St. Louis, MO, USA). Firstly, the fiber was conditioned at 200 °C for 2 min to desorb any contaminants before SPME extraction. Then, it was inserted in the sealed SPME vial with the sample and exposed in the vial headspace. The vial was stirred and incubated at a temperature of 60 °C to accelerate the release of volatiles from the sample matrix into the vial headspace, which were absorbed by the SPME fiber. After 35 min of incubation, the SPME fiber with absorbed volatiles was retrieved from the SPME vial and then loaded into the GC-MS system.

The conditions of GC-MS operation were based on a previous study of Rodriguez et al. (2023) [42]. The GC analysis was performed with an Agilent 7890B installed with a DB 5 fused silica capillary column (30 m × 0.25 mm i.d. × 0.25 µm) and coupled to a 5977A mass selective detector MSD (Agilent Technologies, Lexington, MA, USA). The GC injection port was at 200 °C and in splitless mode. The GC oven temperature was held at 35 °C for 5 min, ramped to 135 °C at a rate of 4 °C/min, then increased by 10 °C/min to 200 °C and maintained for 1.5 min. Helium was the carrier gas at a constant rate of 1 mL/min. The MS detector was operated at an ionization voltage of 70 eV and an ion source temperature of 230 °C. The range of spectra scan was from 45 to 500 *m/z*. The volatile compounds were identified by comparison of the mass spectra and a retention time of their standards and the NIST Library. The concentration was calculated based on the peak areas of ions 69, 93, 121, and 136 *m*/*z*, which are the dominating ions for volatile terpenes.

### 3.4. Sensory Analysis of Odor Perception

A sensory evaluation was conducted in the LSU Agricultural Center Sensory Services Lab which was approved by the Institutional Review Board. The sensory analysis panel consisted of 11 male and 29 female students of Louisiana State University. The preparation of each testing sample was the same as that for the SPME extraction above. After the sample vial was sealed and vortexed completely to form a homogenous solution, it was kept still at room temperature for 30 min before it was evaluated. The odor notes of the major terpenes identified by the GC-MS method above, such as woody, dry-wood, herbal, herb spice, minty, floral, violet, rose, and fruity, were requested to perceive.

### 3.5. Statistical Analysis

Each treatment was carried out in triplicate. All data were statistically analyzed using the SPSS software (version 27; SPSS Inc.; Chicago, IL, USA) and one-way analysis of variance (ANOVA). All data are presented as mean with standard deviation. Significant differences between data were based on *p* < 0.05.

## 4. Conclusions

Like free form terpenes, bound form terpenes could also be significantly present in most plant tissues but not as noticeable as free form terpenes due to their odorless compounds. Compared with polar extraction media, water and ethanol, acetone is the best and food-safe solvent for extracting the bound terpenes in plant tissues. Bound terpenes could be identified and quantified after acid hydrolysis followed by the SPME-GC-MS method. This methodological framework established the groundwork for further works on terpene research beyond the conventional focus on free form terpenes. These further works could include the applications of bound terpenes in food preservation, functional food ingredients, aroma reservoirs with controlled release, cosmetic additives, dietary supplements for health, etc. Also, the developed method and finding of this study could expand the studies on plant physiological function of terpenes from free terpenes to bound terpenes.

However, the types of plant materials evaluated in this study were limited to three fruit peels and corn silk. The direct antimicrobial and bioactive properties from bound terpenes have not been tested and compared with their corresponding free terpenes. Therefore, future research should increase the types of plant materials, including fruit fleshes and vegetables, and compare bound terpenes in fleshes and their byproducts. Also, the research of determining the antimicrobial and bioactive properties of bound terpenes without the interference of free terpenes originally present should be designed and performed. Moreover, the changes in sensory characteristics after bound terpenes are broken down should be studied to provide more information of bound terpenes’ impact on flavor property. Overall, the findings of this study explore the information of bound terpenes and assist in thoroughly understanding terpenes in plants and agricultural products and byproducts. Also, the extraction, characterization, and application of terpenes from agricultural wastes could yield innovative solutions for addressing challenges in food security and environmental conservation.

## Figures and Tables

**Table 1:** Total extraction yields (g/100 g fresh weight) of different samples using three extraction media.

Source	Water	Ethanol	Acetone
Honeydew peel	8.5 ± 0.2 ^a^	8.4 ± 0.2 ^a^	7.9 ± 0.2 ^b^
Watermelon peel	8.4 ± 0.2 ^a^	8.1 ± 0.2 ^a^	7.5 ± 0.2 ^b^
Pineapple peel	8.6 ± 0.3 ^a^	7.1 ± 0.3 ^b^	6.2 ± 0.3 ^c^
Corn silk	7.1 ± 0.5 ^a^	6.4 ± 0.5 ^a^	5.9 ± 0.5 ^b^

Data in the same row with different letters indicate there is a significant difference between them.

**Table 2:** Retention times (RT), retention index (RI), library retention index (LRI), concentrations (μg/g of extract) and odor perception of major terpenes in honeydew peel extracts without (before) and with (after) acid hydrolysis.

			Water		Ethanol		Acetone		Odor
Compound	RI	LRI	Before	After	Before	After	Before	After	Perception
Copaene	1375	1376	-	-	-	-	-	16.0 ± 0.4	Woody, spice ^1^
Epicubebol	1496	1493	-	-	-	-	18.5 ± 0.3 ^a^	16.9 ± 0.3 ^a^	Pleasant aroma, herbal ^2^
Calamenene	1518	1523	-	16.0 ± 0.3 ^a^	12.4 ± 0.3 ^b^	14.4 ± 0.3 ^ab^	41.8 ± 0.4 ^c^	51.6 ± 0.3 ^d^	Herb spice, minty ^3^
Aristol-1(10)-en-9-ol	1623	1642	-	-	-	-	18.5 ± 0.3	-	Woody, floral ^4^
(-)-Isolongifolol, methyl ether	1648	1645	-	-	-	-	17.3 ± 0.3 ^a^	47.1 ± 0.4 ^b^	Woody ^5^
Total			-	16.0	12.4	14.4	96.1	131.6	

Data in the same row with different letters indicate there is a significant difference between them. ^1^: [27] Hu et al., 2019); ^2^: [28] Kirimer et al., 1996; ^3^: [29] Hutchings et al., 2025; ^4^: [30] Jiang et al., 2024; ^5^: [31] Sant’Anna et al., 2007.

**Table 5:** Retention times (RT), retention index (RI), library retention index (LRI), concentrations (μg/g of extract) and odor perception of major terpenes in corn silk extracts without (before) and with (after) acid hydrolysis.

			Water		Ethanol		Acetone		Odor
Compound	RI	LRI	Before	After	Before	After	Before	After	Perception
Geranyl acetone	1459	1453	-	-	-	-	17.5 ± 0.3 ^a^	16.0 ± 0.3 ^a^	Violet, rose, fruity ^1^
γ-Himachalene	1481	1477	-	15.5 ± 0.4 ^a^	-	-	-	15.5 ± 0.4 ^a^	-
Epicubebol	1496	1493	29.3 ± 0.3 ^a^	18.8 ± 0.5 ^b^	-	-	15.3 ± 0.4 ^a^	80.5 ± 0.3 ^c^	Pleasant aroma, Herbal ^2^
Calamenene	1518	1523	-	15.8 ± 0.4 ^a^	-	-	-	14.5 ± 0.5 ^a^	Herb spice, minty ^3^
α-Calacorene	1541	1542	-	17.4 ± 0.4 ^a^	15.0 ± 0.4 ^a^	15.6 ± 0.4 ^a^	-	-	Dry-woody ^4^
Aristol-1(10)-en-9-ol	1623	1692	-	17.6 ± 0.4 ^a^	-	-	-	-	Woody, floral ^5^
Total			29.3	85.1	15.0	15.6	32.8	126.5	

Data in the same row with different letters indicate there is a significant difference between them. ^1^: [29] Hutchings et al., 2025; ^2^: [28] Kirimer et al., 1996; ^3^: [38] Bonikowski et al., 2015; ^4^: [37] Van et al., 2013; ^5^: [30] Jiang et al., 2024.

## Data Availability

Data is contained within the article.
